# Proceedings of the second annual meeting of GenE-HumDi (COST Action 21113)

**DOI:** 10.3389/fgeed.2025.1667329

**Published:** 2025-11-03

**Authors:** María Ortiz-Bueno, Iris Ramos-Hernández, Luis Algeciras-Jiménez, Nechama Kalter, Juan Roberto Rodríguez-Madoz, Jose Bonafont, Rajeevkumar Raveendran Nair, Oliver Feeney, Laura Torella, Lluis Montoliu, Petros Patsali, Claudio Mussolino, Yonglun Luo, Merita Xhetani, Alessia Cavazza, Ayal Hendel, Karim Benabdellah, Carsten Werner Lederer, Francisco J. Molina-Estévez

**Affiliations:** ^1^ GENYO, Centre for Genomics and Oncological Research: Pfizer, University of Granada, Andalusian Regional Government PTS Granada, Granada, Spain; ^2^ Institute of Nanotechnology and Advanced Materials, The Mina and Everard Goodman Faculty of Life Sciences, Bar-Ilan University, Ramat-Gan, Israel; ^3^ Hemato-Oncology Program, Cancer Center Clínica Universidad de Navarra (CCUN), Pamplona, Spain; ^4^ Haemato-Oncology Program, Centre for Applied Medical Research (CIMA), University of Navarra, IdiSNA, Pamplona, Spain; ^5^ DanausGT Biotechnology Ltd., Madrid, Spain; ^6^ Kavli Institute for Systems Neuroscience, NTNU, Trondheim, Norway; ^7^ Research Unit “Ethics of Genome Editing”, Institute of Ethics and History of Medicine, University of Tübingen, Tübingen, Germany; ^8^ DNA and RNA Medicine Division, Center for Applied Medical Research (CIMA), University of Navarra, Pamplona, Spain; ^9^ Department of Molecular and Cellular Biology, National Centre for Biotechnology (CNB-CSIC), Madrid, Spain; ^10^ Centro de Investigación Biomédica en Red de Enfermedades Raras (CIBERER-ISCIII), Madrid, Spain; ^11^ Molecular Genetics of Thalassemia Department, The Cyprus Institute of Neurology & Genetics, Nicosia, Cyprus; ^12^ Institute for Transfusion Medicine and Gene Therapy, Medical Center - University of Freiburg, Freiburg, Germany; ^13^ Center for Chronic Immunodeficiency (CCI), Medical Center — University of Freiburg, Freiburg, Germany; ^14^ Department of Biomedicine, Aarhus University, Aarhus, Denmark; ^15^ Steno Diabetes Center Aarhus, Aarhus University Hospital, Aarhus, Denmark; ^16^ University of Tirana, Department of Biology, Faculty of Natural Science, Tirana, Albania; ^17^ Department of Infection, Immunity & Inflammation, Great Ormond Street Institute of Child Health, University College London, London, United Kingdom; ^18^ Department of Medical and Surgical Sciences for Children and Adults, University of Modena and Reggio Emilia School of Medicine, Modena, Italy; ^19^ Fundación Pública Andaluza para la Investigación Biosanitaria en Andalucía Oriental Alejandro Otero (FIBAO), Granada, Spain

**Keywords:** gene therapy, genome editing, molecular medicine, scientific network, translational medicine, ATMP regulation, harmonization, standards

## Abstract

Genome editing for the treatment of human disease (GenE-HumDi) is an EU-funded COST Action for the development and consolidation of academic, industrial and healthcare feedback networks aiming to accelerate, foster and harmonize the approval of genome-editing (GE) therapies. GenE-HumDi offers mobility grants, supports educational courses, and hosts conferences and meetings to promote synergistic interactions among and across partners active in the discovery, validation, optimization, manufacturing and clinical application of genomic medicines. Furthermore, it provides young and early career scientists with a supportive and world-class environment to foster networking and international collaborations within the GE field. We compiled the proceedings of the second Annual GenE-HumDi Meeting held in Limassol, Cyprus, in 2024. Over three days, renowned experts from the field updated an audience of over 70 GenE-HumDi members and non-member scientists on the latest discoveries and ongoing projects, discussed the status of the field, and identified GenE-HumDi action priorities to advance research and development for GE medicines. Seven focused discussion groups identified gaps in knowledge, standardization and dissemination for new GE tools, delivery methods, safety monitoring, validation for clinical use, and progress in industrial manufacturing and regulatory issues. Simultaneously, publicity about the event itself contributed to outreach and dissemination of GE for human diseases. Therefore, the conclusions of that meeting, summarized here, serve as a compass toward GE application in Europe through coordination, enhanced collaboration and focus on critical developments.

## GenE-HumDi overview

### Form and focus of the Limassol meeting

Recent breakthroughs in GE technologies have created new possibilities for treating diseases through precise and efficient genome modification. The Second Annual Meeting of “Genome Editing for Treatment of Human Diseases” (COST Action 21113: GenE-HumDi), held in Limassol, Cyprus, delved into early achievements by the network, encompassing the creation of new GE tools, advances in efficiency and cell selectivity of delivery tools, progress in cellular and animal models, and updates across a broad spectrum of diseases. The event opened with a public conference followed by two days of in-depth discussion in dedicated working group meetings to address persisting challenges in the field, which include harmonization of assessments and streamlining of regulatory procedures for novel treatments, translation of findings into treatments and company portfolios, and development of an entrepreneurial mindset in young researchers.

From its opening remarks, the meeting emphasized the pivotal role of nurturing the capacities of COST Inclusiveness Target Countries (ITC), such as Cyprus, increasing the pace of new discovery and translation of European gene medicines as well as featuring women leaders in science and industry to inspire and nurture a more balanced distribution of roles in translational GE.

### GenE-HumDi's goal and mission

The ultimate goal of GenE-HumDi is to exploit synergies for faster progress in specific areas and to promote unity, collaboration, and coordination of efforts, for greater resilience and competitiveness of the European GE sector across pharmaceutical and biotech companies, academic institutions, patient advocacy groups and regulatory bodies. Together, we will accelerate the translation of GE technologies for the treatment of human disease.

The mission of COST Action GenE-HumDi is to establish a collaborative and comprehensive research network that unites national centers of excellence in GE. Furthermore, this initiative aims to accelerate the translation of GE technologies into treatments for human diseases by fostering a synergistic and non-overlapping network of basic, translational, and clinical researchers. Our foundational objectives include developing standardized safety assessment protocols, comparing off-target estimation methods, and optimizing GE delivery methods. Therefore, this network not only promotes collaboration through periodic scientific meetings and enhances research quality, but also offers training via organized courses and international exchanges.

In addition, GenE-HumDi focuses on data sharing to improve scientific reproducibility and quality, disseminates research findings to the public to stimulate societal discourse, and engages with legislators and regulators to ensure the efficient and safe advancement of GE within biomedical research. Finally, the initiative seeks to build and enhance the capacity for innovative GE protocols applicable to future clinical trials by supporting training and career development for young researchers willing to contribute to the therapeutic GE field.

**FIGURE 1:**
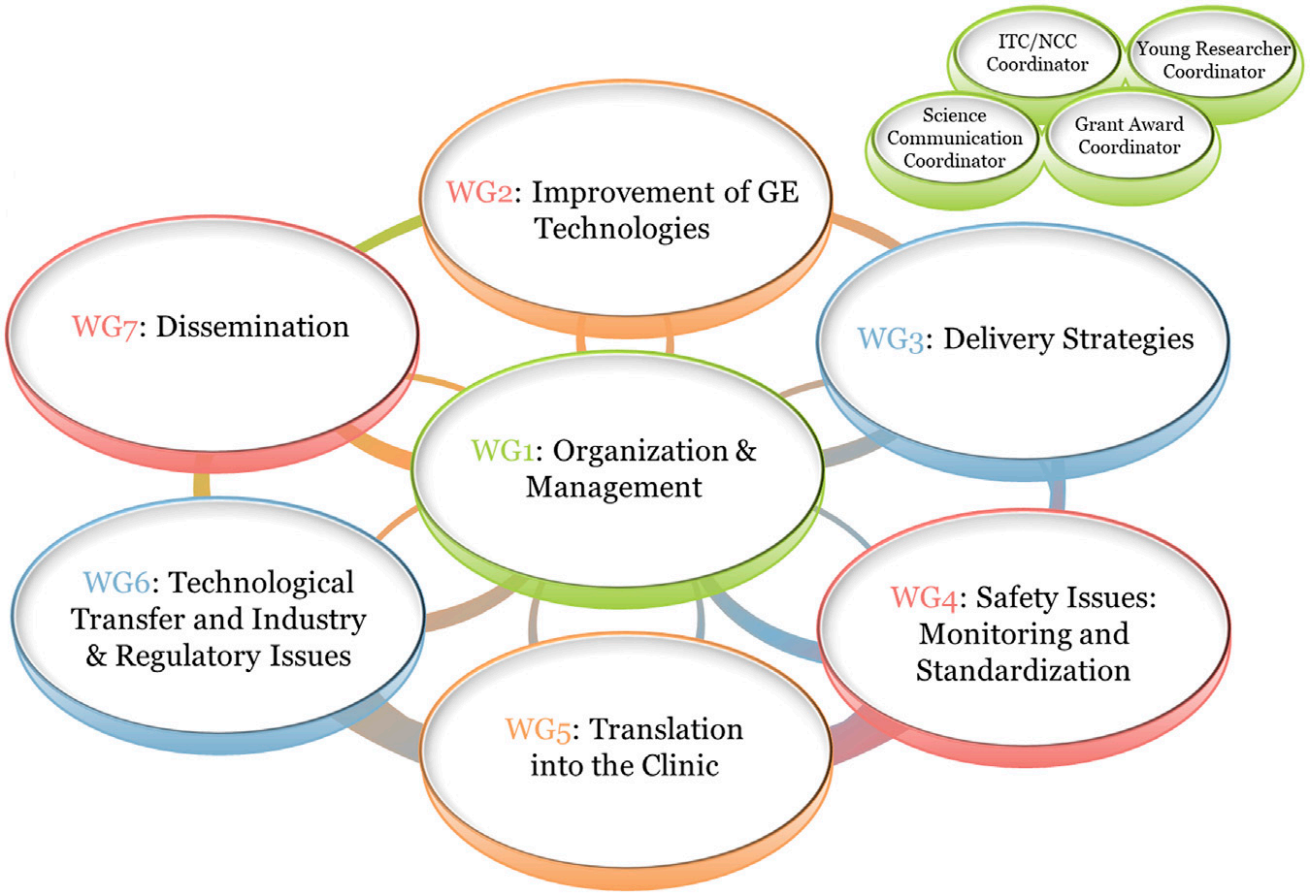
Genome Editing for Treatment of Human Disease Network structure. COST Action 21113 is organized in a way that builds on the aim of coordinating and harmonizing procedures and of promoting new findings and identifying constraints covering all stages of GE medicine development from conception to clinical application, as shown for working groups (WG) 1 through 7. The workload of the network is split across these WGs, with each corresponding to overlapping stages in the process of bringing GE-based ATMPs to patients. Besides, to improve the outreach to and inclusion of specific population targets; ITC, Early Career, Grant Award, and Science Communication coordinators are appointed to promote their specific tasks across all WGs.

Initiated in September 2022, GenE-HumDi (COST Action 21113) is an open network, structured around diverse partners with a track record of excellence in discovery, maturation, progression towards the clinic, regulatory approval and healthcare application of GE technologies. Steadily growing to add further expertise as well as members and organizations in need of training, the network is organized around seven thematic working groups, Figure 1, with logical progression from discovery and validation of new tools and delivery systems (WG2 and WG3), to safety and translation (WG4 and WG5), followed by industry, regulation and dissemination (WG6 and WG7), for altogether, accelerated development and application of new GE-based therapies.

## The GenE-HumDi public conference

### Selected conference papers

This session featuring Europe’s most promising young GE researchers, highlighted advances in safe harbor targeting, hemoglobinopathies and cellular reprogramming. The program included innovations, such as a novel CRISPR/Cas9 platform (KI-Ep) targeting a myeloid-specific safe harbor site in hematopoietic stem and progenitor cells (HSPCs (Ramos-Hernandez et al., 2025)); the validation of an adenine base editor (ABE) adapted for the treatment of β-thalassemia overcoming CRISPR-mediated disruption for correcting the HBB^IVSI-110(G>A)^ mutation (Patsali et al., 2019a; Patsali et al., 2019b); benchmarking distinct ABE tools for the same mutation, identifying an SpG-based editor as key to obtaining a more favorable profile with fewer indel byproducts (Naiisseh et al., 2024); modelling the reversion of the Bernard-Soulier syndrome phenotype by restoring GPIb-V-IX receptor (Martinez-Navajas et al., 2023); the use of dCas9-based tools to convert pancreatic α-cells into insulin-producing β-like cells (Dordevic et al., 2023); and even *in vivo* use of ABE to introduce a protective mutation (−113A>G HPFH) for induction of fetal hemoglobin (HbF). A synopsis of the conference papers and presenters is included in the extended proceedings Supplementary File S1.

### Regulatory and commerzialisation conference session

A session held with translational researchers and leading companies highlighted the vital role for industry as a key asset providing medical devices and clinically scalable tools. The opening topic was how base-edited universal CAR7 T cells hold clinical potential for treating T-cell acute lymphoblastic leukemias and how novel manufacturing protocols reduce turnover times to enable allogeneic use and reducing translocation risks (Chiesa et al., 2023).

The MaxCyte with its GTx electroporation platform showcased its support to more than 70 clinical programs, including the approved therapy for the treatment of hemoglobinopathies CASGEVY^®^. Miltenyi Biotec presented the CliniMACS Prodigy™ closed system that enables academic centers to decentralize manufacturing of CD34^+^ therapies for rare diseases. Cellectis presented non-viral TALEN-edited HSPCs with superior engraftment to address sickle cell mutations without the loss of heterozygosity observed with CRISPR (Moiani et al., 2024).

The speakers collectively demonstrated that base editing and TALEN technologies may reduce off-target effects, and that automated closed systems enhance manufacturing consistency, addressing critical steps toward making these therapies more accessible. These approaches may overcome longstanding barriers in cost, scalability, and safety that hinder widespread clinical adoption of engineered cell therapies. Further details about the papers and presenters are included in the extended proceedings Supplementary File S2.

### New developments conference session

This session demonstrated by key examples from the network how CRISPR methodologies, delivery systems and therapeutic applications hold great promise. Approaches boosting CRISPR/Cas9 efficiency by molecular engineering, like the CCExo system, resulted in increased knockout rates by reducing DNA re-ligation (Lainscek et al., 2022; Shy et al., 2023). They can be delivered by using functionalized lipid nanoparticles (LNPs) for improved mRNA/ribonucleoprotein (RNP) encapsulation. Another innovative alternative for delivery of proteins and nucleic acids is extracellular vesicles (EVs) (Liang et al, 2025). Best EV stability retains up to 90% functionality after one year of storage, with current pharmacokinetic-oriented refinements targeting peptides and cytokine decoys.

Novel tools for *in vivo* HSPC editing using lentiviral vectors (LVs) and virus-like particles (VLPs) evolved from BaEVTR envelopes achieve high transduction of quiescent CD34^+^ cells, with preliminary data on animal models indicating that engineered VLPs increase specificity and enable selective targeting of CD117^+^/CD133^+^ cells, while reducing off-target effects in the liver.

Adressing the challenges of site-specific recombinases (SSRs) for therapeutic genome engineering, new evolved CRE-like recombinases have enhanced DNA target specificity, which is exemplified by a Brec1-ZFN fusion system for potential hemophilia A therapy (Mukhametzyanova et al., 2024). After reviewing several computational tools for CRISPR gRNA design, new AI tools like CRISPRon stand out for their prediction of off-target indel activity (Anthon et al., 2022). Further details about presenters and contents are included in the extended proceedings Supplementary File S3.

### Poster session

Built around young students and early career scientists, the poster session featured *Selected Conference Papers* composed of abstracts evaluated by a scientific committee. The full poster session is summarized in Supplementary Table S2.

## Working groups and plenary discussion

### Network updates and general issues

All WGs conducted thematic discussions on second-year priorities, with outcomes and action plans presented by WG leaders during the concluding session. The meeting’s second phase featured these presentations on Day 2 followed by cross-project action summaries on Day 3. The coordinating group: WG1: Action management and coordination, led by Karim Benabdel Lah, highlighted early successes in fostering young researcher participation through scientific missions, summer schools, and networking opportunities.

### Improvement of GE technology: WG2 session

The WG2: Advances and Challenges in Genome Editing Tools session started with an overview of the safety and efficacy of some current GE toolkit aimed for preclinical use. The tool list included nuclease platforms (ZFNs, TALENs, CRISPR/Cas RNPs), base editors (CBEs, ABEs, GBEs), large-scale integration strategies (HDR, HiTI donors, R2 retrotransposons, and prime editors (PEs)), and some experimental transposase- and recombinase-based systems (FiCAT, PASTE, RNA-Bridges), alongside Cas13a-mediated RNA remodeling. While elevated risks were considered acceptable for certain clinical application, this should be restricted to severe indications. The forum set out the need to improve tissue-specific delivery and efficacy, and to minimize off-target effects for clinical procedures.

Benchmarking PEs unveiled that current utility of PEs is constrained by pegRNA design complexity and the large size of Cas9-RT fusions (Nelson et al., 2022). Therefore, HDR still emerges as a better choice despite concerns of DSB-associated toxicity and off-targets, thus advocating for improving current strategies to enhance repair efficiency.

A concern on immune responses against GE tools was also presented, where in particular pre-existing immunity to commercial proteins may impede universal *clinical* application or reinfusion. This shall be addressed by engineering immune-optimized delivery vectors, personalized patient immune profiling, and developing engineered/natural Cas variants.

Current regulatory guidelines include hurdles for non-clinical assessments and lack disease-specific safety/efficiency thresholds. GenE-HumDi members advocate for better scalability and quality control harmonization as a requirement for cost reduction and global accessibility. Further details about the presenters and views are included in the extended proceedings Supplementary File S4.

### Delivery strategies: WG3 session

GE faces critical challenges delivering GE tools. We need better understanding of non-viral delivery platforms. Lipids, gold and mesoporous silica nanoparticles provide increasing cargo capabilities. However, individual efficiencies vary for ssRNA (mRNAs, miRNAs, and siRNAs), ssDNA (oligodeoxynucleotides (ODNs) and antisense oligonucleotides (ASOs)) and dsDNA (plasmid dsDNA, minicircles, and closed-end donor templates) and are influenced by the protein components. Regarding their toxicity, there is a lack of consensus on suitable benchmarking, as required for harmonization of approaches and standards. Delivery specificity and off-target effects pose three specificity challenges, with limitations regarding tissue, cell, and intracellular destination. Tissue-specific delivery for liver using nanoparticles is efficient but requires dose optimization to avoid toxicity. For muscle targeting, peptide-modified AAVs show promise despite uptake challenges. Last, CNS targeting can draw on engineered AAV capsids and local injections to allow delivery inside the blood-brain barrier (BBB).

Meanwhile, *in vivo* gene therapy for HSC modification can draw on viral vectors, viral-engineered particles and non-viral particles for the delivery of RNA, RNP, and DNA for GE tools. However, clinical HSPC protocols fail to achieve high editing efficiency/specificity and often neglect immunogenicity and inflammatory side effects. Checkpoint gene editing can enhance CAR-T therapies, but targeting hematopoietic stem cells (HSCs) via markers like CD117/CD133 requires careful optimization to prevent unintended effects. While *in vivo* HSC editing promises to simplify manufacturing and lower costs, significant safety concerns regarding immunogenicity and off-target toxicity remain unresolved. Further details concerning the speakers and their views are included in the extended proceedings Supplementary File S4. This session initiated a deeper analysis of the delivery field by the WG3 members, which has since been developed into a full review (Cavazza et al., 2025).

### Safety issues: monitoring and standardization: WG4 session

The Monitoring and standardization session addressed critical challenges for standardizing GE safety evaluation. These included standardization of off-target detection, for which the field urgently requires comparative studies to validate detection tools, as no single method reliably captures all off-target events. Current methods for identifying off-target effects remain fragmented across cell-based, cell-free, and *in silico* approaches, yielding data sets that are hard to reconcile. Functional consequences of editing require improved, clonal analysis during preclinical testing, which could be the key to anticipating adverse effects, as recent clinical setbacks underscored the need to better characterize repair outcomes beyond DNA sequencing. Guide RNA design optimization is still controversial due to the variety of models and methods for optimal gRNA selection. There is an urgent need for rigorous benchmarking based on unified high-quality sequencing data instead of independent datasets. Structural variation detection has long been underestimated due to lack of editor-specific monitoring. Yet, indels and chromosomal rearrangements are frequent short-term outcomes in DNA editing protocols. Even dual nickase strategies, which reduce off-target activity, may increase on-target aberrations, reinforcing the need for better monitoring. Personalized risk assessment requires allele-specific off-target prediction, addressing inter-patient genomic variability, which can critically affect the outcomes and is overlooked by standard methods. Finally, it was discussed that HDR enhancement poses risks, because most HDR-boosting compounds may induce genomic instability in HSPCs and T cells. Accordingly, monitoring using sensitive methods is needed after editing such vulnerable targets. See the extended proceedings Supplementary File S4 for further details. The discussions during this session led to a recent publication addressing the urgent need for standardized guidelines and improved tools for gRNA design, off-target prediction, and activity measurement to ensure the safety and reliability of therapeutic applications (Kalter et al., 2025).

### Translation into the clinic: WG5 session

WG5 explored the formulation of guidelines and documents for the translation of gene editing from bench to bed/market, and discussed standardization of off-target detection, optimization of delivery systems, and assessment of functional outcomes. In doing so the session emphasized the importance of harmonized methodologies and best practices to support reproducibility and safe clinical translation of GE therapies, addressing three critical hurdles in therapeutic development.

Regulatory requirements already pose a burden for preclinical/clinical studies of GE products, covering design, manufacturing, and safety. While an orphan drug designation may facilitate clinical translation, it does so only for rare diseases. And while in the USA, FDA guidelines and requirements are in effect, European consensus on preclinical requirements is lacking despite initiatives such as GenE-HumDi trying to provide a consensus and guidance. Manufacturing scale-up for gene editing maintains editing efficiency but comes with increasing cost barriers. EU grants for GMP validation should always be considered as they significantly aid Investigational New Drug (IND) submission processes and a Clinical Trial Application (CTA) as its EU equivalent. Finally, while patient access barriers and in particular high therapy costs pose a challenge, standard operating procedures (SOPs) and automated closed systems may help reduce production costs. Furthermore, international initiatives (e.g., BioCanRx, Caring Cross) demonstrate successful cost-reduction models, reinforcing the need for centers of excellence, especially for rare disease access. For further details, see the extended proceedings Supplementary File S4.

### Technology transfer and industry and regulatory issues: WG6 session

Technology Transfer and Industry and Regulatory Issues discussed four major hurdles identified by WG6, identified by internal surveys as critical gaps in pediatric applications, equitable access, transparent reporting of data, and harmonization of regulation and data. Addressing these challenges with international guidelines should strengthen Europe’s competitiveness while ensuring GMP compliance. Equitable access is not common practice and would require innovative models, including tiered pricing, intellectual property (IP) pooling and risk-sharing agreements. Use of SOPs could also cut costs to ease access to these therapies. Regarding current pediatric regulatory framework, regulation fails to adequately support therapies for pediatric-specific rare diseases. Worrisome is the weakening of Paediatric Committee (PDCO) oversight, and the need for mechanism-of-action Paediatric Investigational Plans (PIPs) to drive pediatric-specific development rather than adapting adult treatments. Safety reporting is crucial and needs standardization and transparent reporting of all related data, while avoiding unnecessary alarm. The need to further promote patient input into the European Medicines Agency (EMA) Committee for Advanced Therapies (CAT) was considered, as well as the need for unified GE guidelines that address both on-target and off-target effects and incorporate pangenome variability. The overriding challenge of harmonizing regulation and data could in part be addressed by establishing risk thresholds for genetic noise, creating GE tool classifications aligned with global standards, and developing shared databases and human GE registries modeled according to World Health Organization (WHO) germline frameworks to enhance transparency and safety. For details, see the extended proceedings Supplementary File S4.

### Dissemination: WG7 Session

The WG7 session on Dissemination started with a live poll. Collaborative research projects emerged as the top priority, followed by specialized webinars, enhanced digital outreach, and training programs. Answers also advocated conference presentation, multimedia tools and industry partnerships and mentorship programs. A key proposal involved promoting young researchers to present and share GenE-HumDi collaborative work at conferences, such as those organized by the European Society of Gene and Cell Therapy (ESGCT). Further presentations highlighted the need for a forum to connect GenE-HumDi early-career researchers with publishing stakeholders; compiling a proceedings booklet documenting WG outputs as a unified field reference for the COST Action; the need for structured engagement between GenE-HumDi members, WGs, and key stakeholders including patient associations, funders, regulators and pharmaceutical companies; and, last, the key challenge of engaging EU Commission scientific assemblies to update obsolete GE regulations, supported by ARRIAGE consortium partnerships for advocacy. For further details, see the extended proceeding Supplementary File S4.

## Network participation

**FIGURE 2:**
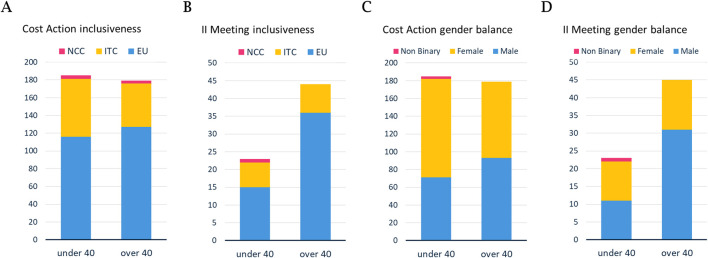
Cost Action and second GenE-HumDi annual meeting demographics. **(A)** Cost Action Members from NCC, ITC, and non-ITC EU in the GenE-HumDi Action and **(B)** corresponding attendance at the conference and meeting, where ITC/NCC reached 30% of young members and approximately 18% of members over 40 years old. **(C)** COST Action 21113 balanced gender representation and **(D)** corresponding gender distribution for the second Annual Meeting, with gender parity for young researchers and approximately 31% women among participants 40 years of age or older.

GenE-HumDi comprises 360 members from 42 countries with a balanced age distribution (Figures 2A,C), with strong representation from “Inclusiveness Target Countries” (ITC) and “Near Neighbour Countries” (NCC) and balanced junior/senior engagement (Figures 2B,D) in the Limassol conference. Gender balance across all career stages aligns with the values of the COST Association. The conference and working group meetings are open to all interested GenE-HumDi member participants, including ITC/NCC representatives and international advisors. In this second Annual Meeting, 18 out of the 42 GenE-HumDi member countries were represented, reflecting the Action’s inclusive policies through slight ITC/NCC overrepresentation (Figures 2C,E) and balanced gender distribution. Presenters and their views are detailed in the Supplemental S1-S4 documents, and a list of all contributors is provided as Supplementary Table S1.

## Data Availability

The original contributions presented in the study are included in the article/Supplementary Material, further inquiries can be directed to the corresponding authors.
