# Supplementary material for: Proceedings of the second annual meeting of GenE-HumDi (COST Action 21113)
Source: Front Genome Ed. 2025 Nov 3;7:1667329. doi: 10.3389/fgeed.2025.1667329 (PMC12620488; doi:10.3389/fgeed.2025.1667329)
Supplement: Supplementary file 1 [file Supplementaryfile1.docx]

**Ortiz-Bueno M, Ramos-Hernández I et al. Supplemental File Session 1**

### Selected Conference Papers

Alessia Cavazza and Laura Torella chaired this session featuring Europe's most promising GE research, highlighting advances in safe harbor targeting, hemoglobinopathies and cellular reprogramming.

**Iris Ramos-Hernández** introduced a novel CRISPR-Cas9 platform targeting hematopoietic stem and progenitor cells (HSPCs), using the CX3CR1 intron as a myeloid-specific safe harbor site. She shared unpublished work showing efficient SFFV-GFP integration via homology-directed repair (HDR). This demonstrated stable expression in HPSCs engrafted in highly immunodeficient NOD scid gamma (NSG) mice without toxicity and with low expression in the most primitive stem cells, improving their safety profile. [1]

**Petros Patsali** validated two approaches for HBBIVSI-110(G>A) thalassemia: CRISPR disruption and SpG adenosine base editor (ABE). The ABE approach showed superior on-target efficiency in patient hematopoietic stem cells (HSCs) and NBSGW mice, maintaining long-term repopulation capacity. This work has been published in part. [2; 3].

**Basma Naiisseh** compared SpRY- and SpG-based ABE tools for correcting the HBBIVSI-110(G>A) mutation, which is prevalent in β-thalassemia. While both ABEs showed similar efficiency, SpG yielded better outcomes with fewer indels. Unexpectedly, editing of the IVSI-107 site affected splicing. This work has been published [4].

**Gonzalo Martínez-Navajas** presented research showing the reversal of the Bernard-Soulier syndrome phenotype by restoring GPIb-V-IX receptor function through lentiviral-mediated GPIX delivery. He also established new disease models using prime-editing specific variants, as reported.[5]

**Marija Đorđević** presented an application of dCas9-Dnmt3a3L-KRAB that achieves conversion of 1% of pancreatic α-cells to insulin-producing cells, resulting in 35% higher insulin output without affecting glucagon levels. This work has been published [6].

**Kyriaki Paschoudi** presented unpublished work demonstrating the use of HDAd5/35++-ABE8e to introduce the -113A>G HPFH mutation in patient-derived CD34+ cells, which successfully increases fetal hemoglobin (HbF) levels in humanized mice without compromising engraftment.

References:

[1] I. Ramos-Hernandez, C. Fuster-Garcia, A. Aguilar-Gonzalez, M.L. Lozano-Vinagre, G. Guenechea-Amurrio, F.J. Sanchez-Luque, M. Goncalves, T. Cathomen, P. Munoz, F.J. Molina-Estevez, and F. Martin, Donor insertion into CX3CR1 allows epigenetic modulation of a constitutive promoter on hematopoietic stem cells and its activation upon myeloid differentiation. Nucleic Acids Res 53 (2025).

[2] P. Patsali, C. Mussolino, P. Ladas, A. Floga, A. Kolnagou, S. Christou, M. Sitarou, M.N. Antoniou, T. Cathomen, C.W. Lederer, and M. Kleanthous, The Scope for Thalassemia Gene Therapy by Disruption of Aberrant Regulatory Elements. J Clin Med 8 (2019).

[3] P. Patsali, G. Turchiano, P. Papasavva, M. Romito, C.C. Loucari, C. Stephanou, S. Christou, M. Sitarou, C. Mussolino, T.I. Cornu, M.N. Antoniou, C.W. Lederer, T. Cathomen, and M. Kleanthous, Correction of IVS I-110(G>A) beta-thalassemia by CRISPR/Cas-and TALEN-mediated disruption of aberrant regulatory elements in human hematopoietic stem and progenitor cells. Haematologica 104 (2019) e497-e501.

[4] B. Naiisseh, P.L. Papasavva, N.Y. Papaioannou, M. Tomazou, L. Koniali, X. Felekis, C.G. Constantinou, M. Sitarou, S. Christou, M. Kleanthous, C.W. Lederer, and P. Patsali, Context base editing for splice correction of IVSI-110 beta-thalassemia. Mol Ther Nucleic Acids 35 (2024) 102183.

[5] G. Martinez-Navajas, J. Ceron-Hernandez, I. Simon, P. Lupianez, S. Diaz-McLynn, S. Perales, U. Modlich, J.A. Guerrero, F. Martin, T. Sevivas, M.L. Lozano, J. Rivera, V. Ramos-Mejia, C. Tersteeg, and P.J. Real, Lentiviral gene therapy reverts GPIX expression and phenotype in Bernard-Soulier syndrome type C. Mol Ther Nucleic Acids 33 (2023) 75-92.

[6] M. Dordevic, P. Stepper, C. Feuerstein-Akgoz, C. Gerhauser, V. Paunovic, A. Tolic, J. Rajic, S. Dinic, A. Uskokovic, N. Grdovic, M. Mihailovic, R.Z. Jurkowska, T.P. Jurkowski, J.A. Jovanovic, and M. Vidakovic, EpiCRISPR targeted methylation of Arx gene initiates transient switch of mouse pancreatic alpha to insulin-producing cells. Front Endocrinol (Lausanne) 14 (2023) 1134478.
