# Supplementary material for: Proceedings of the second annual meeting of GenE-HumDi (COST Action 21113)
Source: Front Genome Ed. 2025 Nov 3;7:1667329. doi: 10.3389/fgeed.2025.1667329 (PMC12620488; doi:10.3389/fgeed.2025.1667329)
Supplement: Supplementary file 2 [file Supplementaryfile3.docx]

**Ortiz-Bueno M, Ramos-Hernández I et al. Supplemental File Session 3**

### New Developments Conference Session

This session covered CRISPR methodologies, delivery systems and therapeutic applications and was chaired by **Francisco Martín Molina**.

**Duško Lainšček** introduced CCExo, a method to boost CRISPR/Cas9 efficiency by recruiting *E. coli* Exonuclease III (EXOIII) to the Cas9/gRNA complex, increasing knockout rates by reducing DNA re-ligation [1]. CCExo could be further enhanced with streptavidin and outperformed Marson’s method[2] ). His ongoing research focuses on delivery using functionalized lipid nanoparticles (LNPs) for improved mRNA/ribonucleoprotein (RNP) encapsulation.

**Dhanu Gupta** introduced therapeutic delivery of proteins and nucleic acids in **extracellular vesicles (EVs**). The strategies presented include endogenous loading, where genetically-modified cells produce pre-loaded EVs, and exogenous loading, involving EV modification after isolation[3]. This presentation highlighted advancements in EV stability, with some formulations retaining 90% functionality after one year of storage, alongside scalable production yielding up to 10¹³ EVs per batch. Pharmacokinetic-oriented refinements include engineering of EV surface modification with targeting peptides and cytokine decoys.

**Luca Biasco** reported ***in vivo* HSPC editing** using lentiviral vectors (LVs) and virus-like particles (VLPs). Pseudotyping VLPs with BaEVTR envelopes improves transduction of quiescent CD34+ cells. In humanized mouse models, VLPs delivered CRISPR machinery resulting in 20% beta-2 microglobulin (**B2M)** knockout after a single dose and up to 50% knockout with repeated administration. Biasco engineered VLPs with Nipah virus-derived glycoproteins, thus increasing specificity and enabling selective targeting of CD117+/CD133+ cells, while reducing off-target effects in the liver.

**Frank Buchholz** addressed the challenges of **site-specific recombinases (SSRs)** for therapeutic genome engineering. Thorough evolution of CRE-like recombinases has enhanced DNA target specificity, which is exemplified by a Brec1-ZFN fusion system for potential hemophilia A therapy [4]. Coupling evolved recombinases with TALE or CRISPR-based DNA-binding domains could accelerate a traditionally years-long process to just months.

Concluding this session, **Jan Gorodkin** (University of Copenhagen, Denmark) reviewed computational tools for **CRISPR gRNA design**, emphasizing CRISPRon[5]. a platform for predicting off-target indel activity. Extensions of this framework include CRISPRon-BE for base editor sgRNA optimization and CRISPRon-Cas12a, which incorporates mRNA and spacer structural data to improve on-target efficiency predictions for Cas12a systems.

**References**

[1] D. Lainscek, V. Forstneric, V. Mikolic, S. Malensek, P. Pecan, M. Bencina, M. Sever, H. Podgornik, and R. Jerala, Coiled-coil heterodimer-based recruitment of an exonuclease to CRISPR/Cas for enhanced gene editing. Nat Commun 13 (2022) 3604.

[2] B.R. Shy, V.S. Vykunta, A. Ha, A. Talbot, T.L. Roth, D.N. Nguyen, W.G. Pfeifer, Y.Y. Chen, F. Blaeschke, E. Shifrut, S. Vedova, M.R. Mamedov, J.J. Chung, H. Li, R. Yu, D. Wu, J. Wolf, T.G. Martin, C.E. Castro, L. Ye, J.H. Esensten, J. Eyquem, and A. Marson, High-yield genome engineering in primary cells using a hybrid ssDNA repair template and small-molecule cocktails. Nat Biotechnol 41 (2023) 521-531.

[3] X. Liang, D. Gupta, J. Xie, E. Van Wonterghem, L. Van Hoecke, J. Hean, Z. Niu, M. Ghaeidamini, O.P.B. Wiklander, W. Zheng, R.J. Wiklander, R. He, D.R. Mamand, J. Bost, G. Zhou, H. Zhou, S. Roudi, H.Y. Estupinan, J. Radler, A.M. Zickler, A. Gorgens, V.W.Q. Hou, R. Slovak, D.W. Hagey, O.G. de Jong, A.G. Uy, Y. Zong, I. Mager, C.M. Perez, T.C. Roberts, D. Carter, P. Vader, E.K. Esbjorner, A. de Fougerolles, M.J.A. Wood, R.E. Vandenbroucke, J.Z. Nordin, and S. El Andaloussi, Engineering of extracellular vesicles for efficient intracellular delivery of multimodal therapeutics including genome editors. Nat Commun 16 (2025) 4028.

[4] L. Mukhametzyanova, L.T. Schmitt, J. Torres-Rivera, T. Rojo-Romanos, F. Lansing, M. Paszkowski-Rogacz, H. Hollak, M. Brux, M. Augsburg, P.M. Schneider, and F. Buchholz, Activation of recombinases at specific DNA loci by zinc-finger domain insertions. Nat Biotechnol 42 (2024) 1844-1854.

[5] C. Anthon, G.I. Corsi, and J. Gorodkin, CRISPRon/off: CRISPR/Cas9 on- and off-target gRNA design. Bioinformatics 38 (2022) 5437-5439.
