# Supplementary material for: Proceedings of the second annual meeting of GenE-HumDi (COST Action 21113)
Source: Front Genome Ed. 2025 Nov 3;7:1667329. doi: 10.3389/fgeed.2025.1667329 (PMC12620488; doi:10.3389/fgeed.2025.1667329)
Supplement: Supplementary file 3 [file Supplementaryfile2.docx]

**Ortiz-Bueno M, Ramos-Hernández I et al. Supplemental File Session 2**

### Regulatory and Commercialization Conference Session

Chaired by **Carla Fuster García** and **Carsten W. Lederer**, this session highlighted recent advances in therapeutic GE with clinical applications. Three researchers and industry leaders presented the following breakthroughs:

The session opened with an academic researcher, **Christos Georgiadis,** from UCL London, advocating for better therapies for B-cell acute lymphoblastic leukemia (B-ALL) and T-cell acute lymphoblastic leukemia (T-ALL), both of which have low survival rates. He introduced the clinical potential of base-edited universal CAR7 T cells for treating T-cell malignancies. His team observed complete remission in three T-ALL patients, and the first patient to be treated maintained the response at two-year follow-up. He also presented an innovative 12-day manufacturing protocol featuring CD7[1] knockout to enable allogeneic use while reducing translocation risks.

We then heard from several industry leaders. **Marianna Romito** (MaxCyte) detailed the company’s GTx electroporation platform that currently supports more than 75 clinical programs, including the approved CASGEVY® therapy for the treatment of hemoglobinopathies. **Eleni Papanikolaou** (Miltenyi Biotec) showcased the CE-marked CliniMACS Prodigy™ system, which enables academic centers to decentralize manufacturing of CD34+ therapies for rare diseases. Closing the industry insights, **Julien Valton** (Cellectis) presented non-viral data on TALEN-edited HSPCs that showed superior engraftment [2].This approach precisely corrected sickle cell mutations without the loss of heterozygosity observed with CRISPR.

The session particularly emphasized safety advancements across platforms. The presenters collectively demonstrated that base editing and TALEN technologies may reduce off-target effects, and that automated closed systems enhance manufacturing consistency, addressing critical steps toward making these therapies more accessible. Simplifying manufacturing through these approaches may overcome longstanding barriers in cost, scalability, and safety that have hindered widespread clinical adoption of engineered cell therapies.

**References**

[1] R. Chiesa, C. Georgiadis, F. Syed, H. Zhan, A. Etuk, S.A. Gkazi, R. Preece, G. Ottaviano, T. Braybrook, J. Chu, A. Kubat, S. Adams, R. Thomas, K. Gilmour, D. O'Connor, A. Vora, W. Qasim, and C.A.R.T.G. Base-Edited, Base-Edited CAR7 T Cells for Relapsed T-Cell Acute Lymphoblastic Leukemia. N Engl J Med 389 (2023) 899-910.

[2] A. Moiani, G. Letort, S. Lizot, A. Chalumeau, C. Foray, T. Felix, D. Le Clerre, S. Temburni-Blake, P. Hong, S. Leduc, N. Pinard, A. Marechal, E. Seclen, A. Boyne, L. Mayer, R. Hong, S. Pulicani, R. Galetto, A. Gouble, M. Cavazzana, A. Juillerat, A. Miccio, A. Duclert, P. Duchateau, and J. Valton, Non-viral DNA delivery and TALEN editing correct the sickle cell mutation in hematopoietic stem cells. Nat Commun 15 (2024) 4965.
