# Supplementary material for: Proceedings of the second annual meeting of GenE-HumDi (COST Action 21113)
Source: Front Genome Ed. 2025 Nov 3;7:1667329. doi: 10.3389/fgeed.2025.1667329 (PMC12620488; doi:10.3389/fgeed.2025.1667329)
Supplement: Supplementary file 4 [file Supplementaryfile4.docx]

**Ortiz-Bueno M, Ramos-Hernández I et al. Supplemental File Session 4**

## Working groups and plenary discussion

### Network Updates and General Issues

All WGs conducted thematic discussions on second-year priorities, with outcomes and action plans presented by WG leaders during the concluding session. The meeting's second phase featured these presentations on Day 2 followed by cross-project action summaries on Day 3. The coordinating group: **WG1: Action management and coordination**, led by Karim Benabdel Lah, highlighted early successes in fostering young researcher participation through scientific missions, summer schools, and networking opportunities.

### Improvement of GE technology: WG2 session

The **WG2: Advances and Challenges in Genome Editing Tools** session, was chaired by **Francisco Martín Molina** and focused on four critical challenges in GE:

**Enhancing Efficacy, Specificity, and Safety. Dimitri Ivancic** outlined current GE toolkits, including nuclease platforms (ZFNs, TALENs, CRISPR-Cas RNPs), base editors (CBEs, ABEs, GBEs), and large-scale integration strategies (HDR, HiTI donors, R2 retrotransposons, and prime editors (PE)). Transposase- and recombinase-based systems (FiCAT, PASTE, RNA-Bridges) were highlighted for their potential in targeted insertion, alongside Cas13a-mediated RNA remodeling. Key discussions centered on **improving tissue-specific efficacy and minimizing off-target effects**.

**Optimizing HDR and Repair Strategies. Claudio Mussolino** compared PE and HDR-based approaches. While PE avoids double-strand breaks (DSBs) and functions well in HSPCs, its utility is constrained by pegRNA design complexity and the large size of Cas9-RT fusions [1]. In contrast, HDR enables flexible template use but carries the risk of DSB-associated toxicity and unintended insertions. Strategies to enhance repair efficiency include drug-modulated DNA repair and DSB-tethered repair templates.

**Overcoming Immune Recognition. Julian Ceron** highlighted the occurrence of immune responses against GE tools, in particular pre-existing immunity to SpCas9, SaCas9 and dCas9 proteins, which **may impede *in vivo* application** and reinfusion regimens of these tools. He called for solutions including immune-optimized delivery vectors, patient-specific immune profiles, and engineered/natural Cas variants to minimize cross-reactivity.

**Clinical Translation Challenges. Laura Torella** reviewed regulatory hurdles, including FDA guidelines for non-clinical assessments and disease-specific safety/efficiency thresholds. With the first CRISPR therapy approved and more than 50 GE trials underway, scalability, quality control harmonization and cost reduction remain vital for global accessibility.

### Delivery Strategies: WG3 Session

Next, the **WG3: Genome Editing Delivery Challenges session**, chaired by **Yonglun Luo**, addressed three critical challenges in delivering GE tools:

**Delivery System Efficiency**

**Álvaro Somoza** and **Manuel Gonçalves** reviewed non-viral delivery platforms. Regarding cargo capacity, LNPs carry 2-3 mRNA molecules per particle, while mesoporous silica nanoparticles achieve higher loading capacity (95 µg siRNA/mg). Gold nanoparticles show intermediate efficiency (100-200 surface-adsorbed siRNA molecules). Individual efficiencies vary based on their design and the type of cargo, which encompasses ssRNA (mRNAs, miRNAs, and siRNAs), ssDNA (oligodeoxynucleotides (ODNs) and anti-sense oligonucleotides (ASOs)) and dsDNA (plasmid dsDNA, minicircles, and closed-end donor templates) as nucleic acid components, and ZFN, TALEN and Cas9/Cas12a, base/prime editors, transposases and recombinases as protein components. Current optimization efforts focus on tissue-selective delivery and cargo capacity improvements for different payloads.

**Yonglun Luo** identified three specificity levels with limitations regarding **specificity and off-target effects:** tissue, cellular, and intracellular. Furthermore, despite the saturation of gRNA design tools there is no consensus on algorithms, procedures or controls. Regarding genotoxicity assessments, bioinformatic approaches overestimate gRNA off-targets. In an arbitrary gRNA example presented, from 2,052 off targets called out *in silico*, experimental validation in HEK293T cells confirmed only a few and with very poor efficiency, illustrating the need for standardized evaluation across cell types, particularly for primary cells. Benchmarking initiatives are required for harmonization of approaches and standards, yet current platforms are difficult to cross-compare due to lack of overlapping targeted cell types.

**Lorea Blázquez** discussed **tissue-specific delivery strategies,** focusing on the following target tissues: Liver: here, GalNAc-coated nanoparticles improve delivery but require dose optimization to avoid toxicity; Muscle: peptide-modified AAVs show promise despite uptake challenges; CNS: engineered AAV capsids and local injections address blood-brain barrier (BBB) penetration limitations. Overall, while targeted delivery methods are advancing, each tissue presents unique challenges that require tailored strategies to optimize efficiency and minimize adverse effects.

**Annarita Miccio** gave an update on *ex vivo* gene therapy for HSC modification, which includes viral (LV/VLP, AAV, adenoviral vectors) and non-viral (LNP, polymeric NP, gold NP) delivery of RNA, RNP, and DNA-editing tools. However, current HSPCs protocols lack competitive high editing efficiency/specificity and, besides, they still need to improve their immunogenicity and inflammatory side effects. Some CAR-T therapies benefit from checkpoint gene editing, though target specificity (CD117/CD133 for HSCs) requires careful optimization to minimize unintended effects. Efforts are ongoing to perfect HSC editing *in vivo*; these efforts are based on the premise that *in vivo* HSC editing may simplify manufacturing, accelerate patient benefits and lower costs, but there are unresolved concerns due to immunogenicity, safety and off-target toxicity.

This session started a deeper analysis of the delivery field by the WG3 members which matured in a thorough revision already available.[2]

### Safety issues: Monitoring and Standardization: WG4 Session

**Ayal Hendel** and **Ciaran Lee** led **WG4: Safety issues: Monitoring and Standardization** in addressing six critical challenges for standardizing GE safety evaluation:

**Ciaran Lee** presented **off-target detection standardization**. Current methods for identifying off-target effects remain fragmented across cell-based, cell-free, and *in silico* approaches, yielding data sets that are hard to reconcile. The field urgently requires comparative studies to validate detection tools, as no single method reliably captures all off-target events.

**Ayal Hendel** introduced the **functional consequences of editing.** Recent clinical setbacks in sickle cell disease trials (e.g., Graphite Bio, Novartis) and PCSK9 editing for hypercholesterolemia (e.g., Verve Therapeutics) underscore the need to better characterize repair outcomes beyond DNA sequencing. Improved clonality analysis during preclinical testing could be the key to anticipating adverse effects.

**Jan Gorodkin** emphasized the importance of **gRNA design optimization**. Predictive tools require high-quality sequencing data to build computational models (machine learning). Recent literature has published a variety of such models, but these lack consensus on optimal gRNA selection methods, with most being inadequately validated against independent datasets, urging the need for rigorous benchmarking.

**Carla Fuster** highlighted advances and challenges in **structural variation detection**. All Cas9- and even base-editing tools induce unexpected indels and chromosomal rearrangements. While dual nickase strategies reduce off-target activity, they may increase on-target aberrations, thus necessitating editor-specific monitoring.

**Nechama Kalter** introduced **personalized risk assessment** demonstrating how CRISPECTOR2.0 enables allele-specific off-target prediction, addressing inter-patient genomic variability that standard methods overlook.

**Giandomenico Turchiano** warned about **HDR enhancement risks**, emphasizing that most HDR-boosting compounds may induce genomic instability in HSPCs and T cells. New “high-resolution CAST-seq” methods now detect off-targets with 20-fold greater sensitivity.

### Translation into the Clinic: WG5 Session

**Alessia Cavazza** chaired the WG5 session on **translation challenges for GE therapies**, addressing three critical hurdles in therapeutic development:

**Paula Río** presented **regulatory roadmap** complications. Recent FDA guidelines outline requirements for preclinical/clinical studies of GE products, covering design, manufacturing, and safety (on/off-target effects, immunogenicity). While Orphan Drug Designation accelerates rare disease therapy development through incentives like market exclusivity, a European consensus on preclinical requirements is lacking, with initiatives like GenE-HumDi providing crucial guidance.

**José Bonafont** elaborated **manufacturing scale-up** considerations. Transitioning from R&D to Good Manufacturing Practice (GMP) production maintains editing efficiency but comes with increasing cost barriers. Upon successful approval, autologous therapies show limited cost reduction potential versus allogeneic approaches. EU grants for GMP validation should always be considered as they significantly aid Investigation New Drug (IND) submission processes.

**Alessia Cavazza** emphasized **patient access barriers**. Therapy costs vary dramatically between academic centers (far lower cost, longer waiting times) and contract development and manufacturing organizations (CDMOs). Standard operating procedures (SOPs) and automated closed systems may help reduce production costs by ~30%. Furthermore, international initiatives (e.g., BioCanRx, Caring Cross) demonstrate successful cost-reduction models. Hence, building collaborative networks and centers of excellence is essential for rare disease access.

### Technology Transfer and Industry & Regulatory Issues: WG6 Session

Chaired by **Carsten W. Lederer**, the **WG6: Technology Transfer and Industry & Regulatory Issues** session presented 4 major hurdles identified by the WG:

**Dr. Lederer** presented pre-meeting surveys identifying critical gaps in pediatric applications, equitable access, safety and transparent reporting, and an overall need for harmonization of regulation and data standards. Proposed solutions included Advanced Therapy Medicinal Product (ATMP) workshops, white papers and international guidelines to strengthen Europe’s competitiveness while ensuring GMP compliance.

**Oliver Feeney** elaborated on **equitable access**, emphasizing that financial-ethical conflicts require innovative models, including tiered pricing, intellectual property (IP) pooling and risk-sharing agreements. Balancing centralized excellence centers with decentralized manufacturing could reduce regional disparities, while standardization of platform technologies through SOPs may cut approval timelines by 30-50%.

**Alejandro Barquero** elaborated on the need for a **pediatric regulatory framework**. Current pediatric regulation fails to adequately support therapies for pediatric-specific rare diseases. Several issues were pointed out, including replacement of EU pharmaceutical binding regulations with non-binding directives, the weakening of “Paediatric Committee” (PDCO) oversight, and the need for mechanism-of-action “Paediatric Investigational Plans” (PIPs) to drive pediatric-specific development rather than adapting adult treatments. Simultaneously, new ethical and regulatory frameworks must be developed for emerging applications such as fetal and *in utero* genome editing. This situation urges the creation of tailored frameworks for rare diseases and prenatal interventions where current regulations are silent or inadequate.

**Carla Fuster** examined crucial safety reporting challenges for genome-editing therapies, emphasizing the need for transparent reporting of off-target mutations while avoiding unnecessary alarm. It’s imperative to standardize assays and rigorously assess potentially disease-causing indels, enabling personalized monitoring of GE outcomes. However, we still face the challenge of populational genomic variations introducing unpredictability in gRNA design, safety, and therapeutic efficacy. The need to further promote patient input into the European Medicines Agency (EMA) Committee for Advanced Therapies (CAT) was considered. The discussion concluded that maintaining the ongoing dialogue between regulators and patient advocacy groups remains essential for advancing GE therapies responsibly.

**Lluis Montoliu** concluded the session by emphasizing the need for unified GE guidelines that address both on-target and off-target effects and incorporate pangenome variability. Key recommendations included establishing risk thresholds for genetic noise, creating GE tool classifications that are aligned with global standards, and developing shared databases and human GE registries modeled according to World Health Organization (WHO) germline frameworks to enhance transparency and safety. These efforts aim to build consistent regulatory standards and collaborative platforms.

### Dissemination, WG7 Session

**Javier Molina Estévez** chaired the **WG7 session on Dissemination**, in which **Merita Xhetani** presented key findings from a digital poll of early-career members. Collaborative research projects emerged as the top priority (22%), followed by specialized webinars (18%), enhanced digital outreach (17%), and training programs (16%). Answers also advocated for conference presentations (e.g., ESGCT events) and multimedia tools to boost visibility. Industry partnerships and mentorship programs each received 13% support. When asked about concrete tools, respondents suggested leveraging mass media, short videos and collaborations with established societies. A key proposal involved promoting young researchers to present and share GenE-HumDi collaborative work at conferences such as those organized by the European Society of Gene and Cell Therapy (ESGCT).

**Josip Madunić** proposed the organization of a forum to connect GenE-HumDi early-career researchers with publishing stakeholders overlapping with European meetings for optimal engagement of both parties.

**Neli Kachamakova-Trojanowska** advocated for compiling a proceedings booklet documenting WG outputs as a unified field reference for the COST Action. This would be favored over a special journal issue owing to logistical challenges in journal selection and cross-WG coordination.

**Luka Bockor** identified the need for structured engagement between GenE-HumDi members, WGs, and key stakeholders including patient associations, funders, regulators and pharmaceutical companies. The discussion prioritized patient groups as a strategic gateway to indirectly involve regulators and funders, while proposing increased conference presence to engage healthcare providers and amplify impact.

**Lluis Montoliu** outlined WG7's key challenge: engaging EU Commission scientific assemblies to update obsolete GE regulations, noting unchanged regulations since the 1997 Oviedo Convention, despite tremendous technological advances. He highlighted the FDA's 2024 GE guidelines as contrasting with EMA's framework gap, proposing to leverage ARRIAGE consortium partnerships for advocacy. The COST Action must first establish an internal consensus and draft a unified position memorandum before EU engagement.

**References:**

[1] J.W. Nelson, P.B. Randolph, S.P. Shen, K.A. Everette, P.J. Chen, A.V. Anzalone, M. An, G.A. Newby, J.C. Chen, A. Hsu, and D.R. Liu, Engineered pegRNAs improve prime editing efficiency. Nat Biotechnol 40 (2022) 402-410.

[2] A. Cavazza, F.J. Molina-Estevez, A.P. Reyes, V. Ronco, A. Naseem, S. Malensek, P. Pecan, A. Santini, P. Heredia, A. Aguilar-Gonzalez, H. Boulaiz, Q. Ni, M. Cortijo-Gutierrez, K. Pavlovic, I. Herrera, B. de la Cerda, E.M. Garcia-Tenorio, E. Richard, S. Granados-Principal, A. Lopez-Marquez, M. Kober, M. Stojanovic, M. Vidakovic, I. Santos-Garcia, L. Blazquez, E. Haughton, D. Yan, R.M. Sanchez-Martin, L. Mazini, G.G. Aseguinolaza, A. Miccio, P. Rio, L.R. Desviat, M. Goncalves, L. Peng, C. Jimenez-Mallebrera, F.M. Molina, D. Gupta, D. Lainscek, Y. Luo, and K. Benabdellah, Advanced delivery systems for gene editing: A comprehensive review from the GenE-HumDi COST Action Working Group. Mol Ther Nucleic Acids 36 (2025) 102457.
